# Fatal outcome of autosomal recessive polycystic kidney disease in neonates with recessive *PKHD1* mutations

**DOI:** 10.1097/MD.0000000000020113

**Published:** 2020-05-08

**Authors:** Jiwon Jung, Go Hun Seo, Yoo-Mi Kim, Young Mi Han, Ji Kwon Park, Gu-Hwan Kim, Joo Hoon Lee, Young Seo Park, Byong Sop Lee, Ellen Ai-Rhan Kim, Pil-Ryang Lee, Beom Hee Lee

**Affiliations:** a Department of Pediatrics, Asan Medical Center Children's Hospital, University of Ulsan College of Medicine, Seoul; b 3billion, Inc.; c Department of Pediatrics, Chungnam National University School of Medicine, Chungnam National University Hospital, Daejeon; d Department of Pediatrics, Pusan National University Children's Hospital, Pusan; e Department of Obsteterics, Gyeongsang National University Changwon Hospital, Gyeongsang National University School of Medicine, Changwon; f Medical Genetics Center, Asan Medical Center Children's Hospital; g Department of Obstetrics, Asan Medical Center, University of Ulsan College of Medicine, Seoul, South Korea.

**Keywords:** autosomal recessive polycystic kidney disease, mutation, *PKHD1* gene, prenatal diagnosis

## Abstract

Autosomal recessive polycystic kidney disease (ARPKD) is the most common inherited childhood-onset renal disease, with underlying ciliopathy, and varies widely in clinical severity. The aim of this study was to describe the most severe form of ARPKD, with a fatal clinical course, and its association with mutations in polycystic kidney and hepatic disease 1 (fibrocystin) (*PKHD1*). Clinical, imaging, pathological, and molecular genetic findings were reviewed in patients prenatally affected with ARPKD and their families.

Five unrelated Korean families, including 9 patients, were analyzed. Among the 9 patients, 2 fetuses died in utero, 6 patients did not survive longer than a few days, and 1 patient survived for 5 months with ventilator support and renal replacement therapy. A total of 6 truncating mutations (all nonsense) and 4 missense mutations were detected in a compound heterozygous state, including 4 novel mutations. The most severe phenotypes were shared among all affected patients in each family, irrespective of mutation types.

Our data suggest a strong genotype–phenotype relationship in ARPKD, with minimal intra-familial heterogeneity. These findings are important for informing future reproductive planning in affected families.

## 1 Introduction

Autosomal recessive polycystic kidney disease (ARPKD; MIM 263200) is one of the most common childhood-onset ciliopathies and is characterized by bilateral renal cystic disease and congenital hepatic fibrosis. Although the estimated incidence of ARPKD is approximately 1:10,000 to 1:40,000, it could be higher, since the most severely affected newborns may not be diagnosed, due to early death within the first few days of life.^[1–3]^ Approximately 40% of patients with ARPKD present with enlarged, hyperechogenic kidneys, poor corticomedullary differentiation, and oligohydramnios, detected by prenatal sonography in the 21st to 24th week of gestation, which often leads to postnatal death, secondary to pulmonary hypoplasia.^[4,5]^

ARPKD is caused by recessive mutations of the polycystic kidney and hepatic disease 1 (*PKHD1*) gene on chromosome 6p21. *PKHD1* encodes fibrocystin, a 4074 amino acid protein expressed in the primary cilia of the renal collecting duct, bile ducts, epithelial cells, and pancreas.^[6–8]^ Deficiency of fibrocystin leads to disordered terminal differentiation, dilatation, and fibrosis of the renal collecting and intrahepatic biliary ducts.^[9]^ To date, more than 750 *PKHD1* mutations have been reported, among which approximately 60% are truncating and 40% are missense (http://www.humgen.rwth-aachen.de).

Patients with ARPKD exhibit a wide spectrum of phenotypes, depending on age at presentation and mutation types.^[2–5,9,10]^ The majority of cases are identified at birth or in late pregnancy, and the most severely affected fetuses display a “Potter” phenotype, with bilaterally enlarged kidneys, pulmonary hypoplasia, characteristic facies, and sometimes contracted limbs with club feet.^[11]^ Mortality from ARPKD is highest within the 1st month after birth, reaching 50%.^[4]^ Patients with 2 truncating mutations in *PKHD1* have almost 100% lethal outcomes, while some patients with either 1 or 2 missense mutations may survive through the neonatal period^[2,3,5,9,10]^; however, this rule does not always apply, as some missense mutations can have consequences as severe as those of truncating alterations.^[3,9]^

Here we present a series of neonates and fetuses, severely prenatally affected with ARPKD, with the aim of determining the influence of specific *PKHD1* genotypes on the most severe ARPKD phenotypes.

## 2 Materials and methods

### 2.1 Patients

From April 2012 to July 2017, 30 patients suspected to have ARPKD and their families were referred to the Medical Genetics Center, Asan Medical Center, Seoul, Korea, for genetic testing of *PKHD1*. Among these families, subjects with a history of abnormal renal fetal ultrasonography (US) findings, including kidney abnormalities and/or severe oligohydramnios, resulting in fetal demise, were included in the current study.

Patient medical records were reviewed for the following information: family obstetric history, prenatal US findings, and autopsy findings of deceased fetuses. The study was approved by the Institutional Review Board of the Asan Medical Center, Seoul, Korea.

### 2.2 Analysis of the *PKHD1* gene

Genomic DNA was isolated from peripheral blood using a PUREGENE DNA isolation kit (Qiagen, Hilden, Germany). Sequences of 67 *PKDH1* exons and their intronic flanking sequences were amplified by PCR using 75 sets of primers designed using primer3 cgi v.3.0 (Whitehead Institute, http://bioinfo.ut.ee/primer3–0.4.0/) based on sequences from GenBank, accession number: NT_007592.15. DNA sequencing was performed with the same primers used for PCR and a BigDye Terminator V3.1 Cycle Sequencing Ready reaction kit (Applied Biosystems, Foster City, CA), according to the manufacturer's instructions. Electrophoresis and analysis of the reaction mixtures were conducted using an ABI 3130xl Genetic analyzer (Applied Biosystems).

### 2.3 In silico analysis

We used Polyphen – 2 (http://genetics.bwh.harvard.edu/pph2/index.shtml) and SIFT (Sorting Intolerant From Tolerant, https://sift.bii.a.-star.edu.sg) for the in silico analysis of functional effect of missense mutations. In Polyphen – 2, mutations are qualitatively assessed as a score of 0 to 1 based on the protein structure, function, and evolutionary conservation; from 0 being totally benign to a higher score possessing more probability of damaging function. SIFT also provides qualitative scores 1 to 0 based on the sequence homology and the physical properties of amino acids; from 1 predicting benign influence to 0 associated with damaging of the function.

## 3 Results

During the study period, 9 deceased fetuses or neonates from 5 unrelated families were diagnosed with ARPKD due to *PKHD1* mutations. The clinical features of the 9 patients are summarized in Table 1.

**Table 1:**
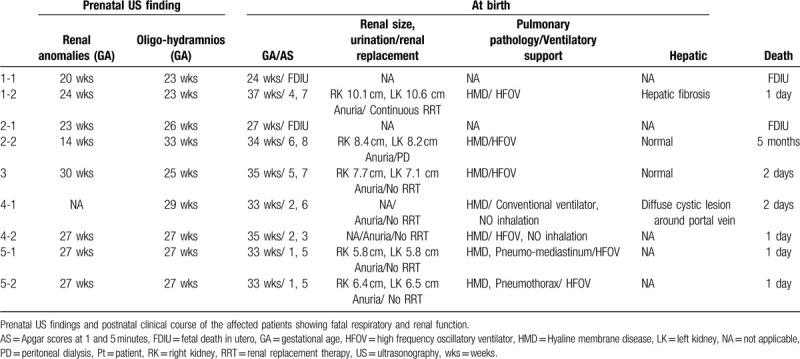
Clinical features of patients with severe ARPKD.

### 3.1 Family 1: Patients 1-1 and 1-2

**Figure 1:**
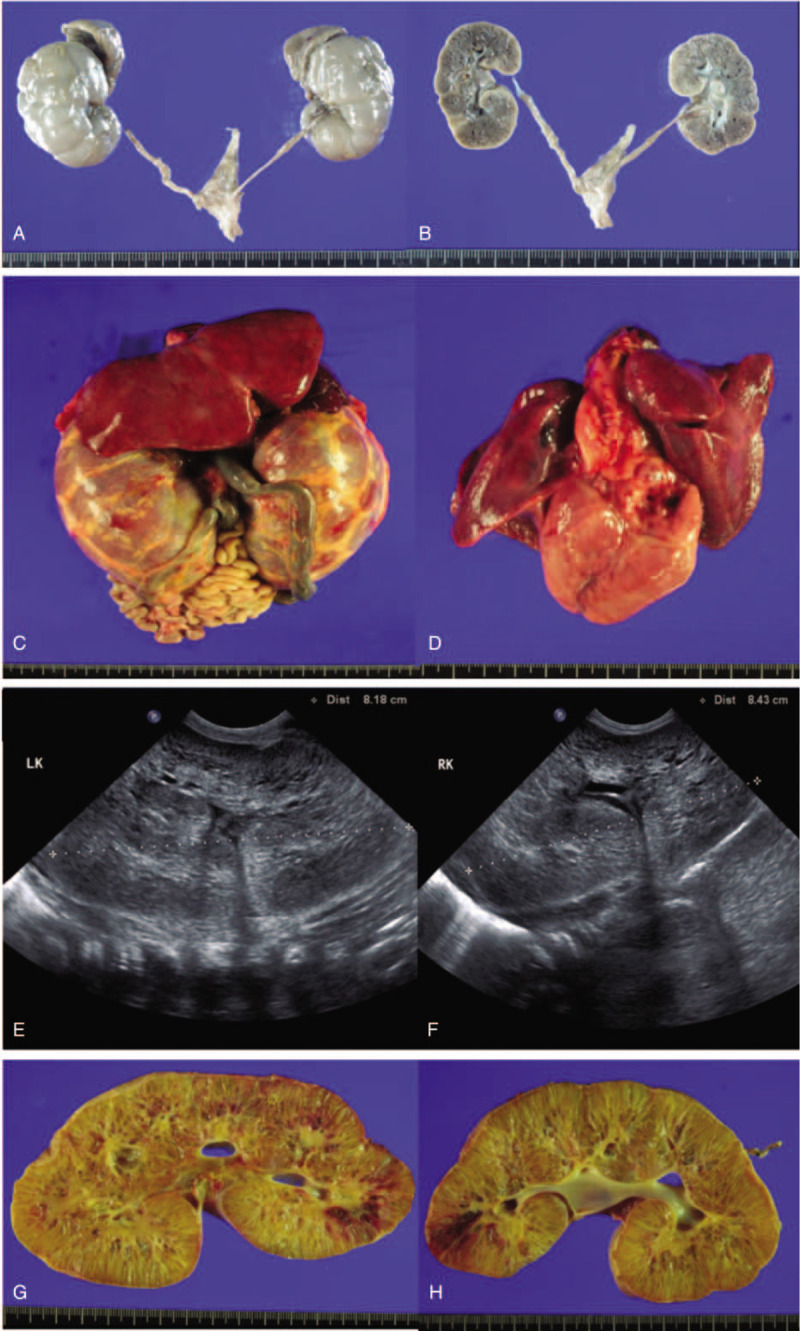
Findings from Family 1 and Family 2. Autopsy images of kidney from patient 1-1. A, Kidneys are enlarged, and their external surfaces are lobulated. B, On hemisection, surfaces are spongy and show multiple small cysts, measuring up to 0.2 cm; the cysts are filled with clear serous fluid. The corticomedullary junction is faintly demarcated. Autopsy images from patient 1-2. C, Gross anatomy showing enlargement of both kidneys. D, Hypoplasia of both lungs; hyaline membrane disease was detected on microscopic examination. Ultrasound images and autopsy images from patient 2-2 after birth. E, F, Both kidney enlargement (left 8.18 mm, right 8.43 mm) with loss of normal corticomedullary differentiation and increased cortical echogenicity, due to multiple interfaces associated with dilated small medullary microcysts. G, Gross appearance of left kidney with multiple variable—sized linear or round cysts in entire kidney (up to 0.8 cm in greatest dimension). H, Gross appearance of right kidney also with multiple vertical linear or round cysts in entire kidney (up to 0.5 cm in greatest dimension).

Patient (Pt) 1-1 was from the first pregnancy of the family. Prenatal US at gestational age (GA) 20 weeks revealed bilateral enlarged hyperechoic kidneys. At GA 23 weeks, severe oligohydramnios was detected (amniotic fluid index [AFI]: 63 mm). Pt 1-1 died at GA 24 weeks, and autopsy under the parents’ formal consent revealed a female fetus with bilateral cystic kidneys and hepatic fibrosis; however, no genetic evaluation was conducted. Autopsy findings of patient 1-1 are presented in Fig. 1A–B.

Pt 1-2, a male from the family's second pregnancy, was born at GA 37 weeks. At GA 22 weeks, oligohydramnios was detected (AFI: 82 mm), and amnioinfusion was carried out 4 times from GA 23 to 31 weeks. At birth, Apgar scores were 4 at 1 minute and 7 at 5 minutes. The patient required immediate intubation and ventilator support using a high frequency oscillatory ventilator (HFOV). Continuous renal replacement therapy (CRRT) was applied because of anuria. Kidney US showed bilateral kidney enlargement, with loss of normal corticomedullary differentiation and microcysts (right kidney: 10.1 cm; left kidney: 10.6 cm). Pt 1-2 died of respiratory failure secondary to pulmonary hypoplasia on the 1st day of life. Autopsy revealed ARPKD with hepatic fibrosis and hyaline membrane disease of both lungs. Autopsy findings of patient 1-2 are presented in Figure 1C–D.

Genetic analysis of a peripheral blood sample revealed a missense mutation, c.274C>T (p.Arg92Trp), in exon 4, and a nonsense mutation, c.2770C>T (p.Gln924∗), in exon 26, confirming the diagnosis of ARPKD with *PKHD1* mutation. The missense mutation, c.274C>T (p.Arg92Trp) was analyzed with in silico investigations and predicted to have a damaging effect on the protein function. The patient's mother was a heterozygous carrier of c.274C>T (p.Arg92Trp), and the patient's father was a heterozygous carrier of c.2770C>T (p.Gln924∗). Patient 1-2 and the brief family history was previously reported as a newly detected *PKHD1* mutation as a case report in 2015.^[12]^

### 3.2 Family 2: Patients 2-1 and 2-2

Pt 2-1 was from the second pregnancy of the family. Prenatal US at GA 23 weeks showed bilateral enlarged hyperechoic kidneys (both 3.7 cm). Oligohydramnios was first detected at GA 26 weeks (AFI: 72.9 mm), rapidly aggravated (AFI: 30 mm), and the fetus died at GA 27 weeks. Autopsy under the parents’ formal consent revealed a male fetus with bilateral enlarged polycystic kidneys and hepatic fibrosis.

Pt 2-2, a male from the family's third pregnancy, was born at GA 34 weeks and 2 days. Oligohydramnios was first detected at GA 30 weeks. The patient received 350 mL amnioinfusion at GA 32 + 4 weeks. At birth, Apgar scores were 6 at 1 minute and 8 at 5 minutes. The patient required immediate intubation and ventilator support with HFOV for 1 week, and subsequently with a conventional ventilator. Postnatal kidney US showed bilateral kidney enlargement, with loss of normal corticomedullary differentiation (right kidney: 8.43 cm; left kidney: 8.18 cm). Concentric hypertrophy of both ventricles due to hypertension was detected by echocardiography at 2 months of age. The patient underwent peritoneal dialysis because of anuria after birth. Hepatic US revealed biliary micro-hamartoma and ductal plate malformation. The patient died of hypotensive cardiogenic shock at 5 months of age. Genetic testing of the *PKHD1* gene using a peripheral blood sample from Pt 2-2 revealed a previously unreported, nonsense mutation, c.68421G>A (p. Trp2280∗), in exon 42, and another nonsense mutation, c.11074C>T (p. Arg3692∗), in exon 61. His parents refused the carrier testing for the *PKHD1* mutations.

Sonographic findings and autopsy findings of patient 2-2 are presented in Fig. 1E–H.

### 3.3 Family 3: Patient 3

Pt 3 was a male from the second pregnancy of the family. Prenatal US at GA 25 weeks showed oligohydramnios with bilateral enlarged kidneys. The patient underwent 3 rounds of amnioinfusion at GA 25 + 2 weeks, 28 + 2 weeks, and 32 + 5 weeks. At birth, Apgar scores were 5 at 1 minute and 7 at 5 minutes. The patient required intubation and ventilator support with HFOV. Kidney US showed bilateral kidney enlargement with loss of normal corticomedullary differentiation (right kidney: 7.7 cm; left kidney: 7.0 cm) and ureteral dilatation. Even with full respiratory support and nitric oxide inhalation, respiratory failure progressed, and the patient became anuric. The patient died due to respiratory failure at 2 days old. Peripheral leukocytes were used for genetic analysis of *PKHD1*, revealing one novel nonsense mutation, c.8208G>A (p.Trp2736∗), in exon 52, and 1 missense mutation, c.9719G>A (p.Arg3240Gln), in exon 58. After in silico analysis, the latter mutation was predicted to have a damaging effect on the protein function. His parents refused carrier testing for the mutations.

### 3.4 Family 4: Patients 4-1 and 4-2

Pt 4-1 was a male, born from the family's first pregnancy. Anhydramnios with bilaterally enlarged hyperechoic kidneys was detected by US at GA 29 weeks, leading to 5 rounds of amnioinfusion. At birth, Apgar scores were 2 at 1 minute and 6 at 5 minutes. Due to lung hypoplasia, he needed intubation with ventilator support, NO gas inhalation, and surfactant inhalation, which did not reverse the course of respiratory failure. The patient was anuric and efforts at peritoneal dialysis were unsuccessful. The patient expired on the second postnatal day due to respiratory failure.

Pt 4-2 was also a male from the second pregnancy of the family, who presented with enlarged kidneys with oligohydramnios at GA 27 weeks, and received 1 round of amnioinfusion. Patient was born at GA 34 + 6 weeks, and expired within 3 hours due to respiratory failure with recurrent pneumothorax secondary to lung hypoplasia, on the 1st postnatal day.

Analysis of cord blood DNA from Pt 4-1 later revealed two heterozygous nonsense mutations of *PKHD1*, c.982C>T (p.Arg328∗), and the novel mutation, c.10228C>T (p.Gln3410∗), in exons 14 and 61, respectively. Analysis of amniocyte from Pt 4-2 revealed the same 2 nonsense mutations in *PKHD1*. The mother of Pt 4-1 and 4-2 was a heterozygous carrier of c.982C>T (p.Arg328∗), and the father was a heterozygous carrier of c.10228C>T (p.Gln3410∗). The family underwent genetic counseling with cord villous sampling during a subsequent pregnancy, giving birth to a non-affected child with normal sequence at the relevant sites of the *PKHD1* gene.

### 3.5 Family 5: Patients 5-1 and 5-2

**Figure 2:**
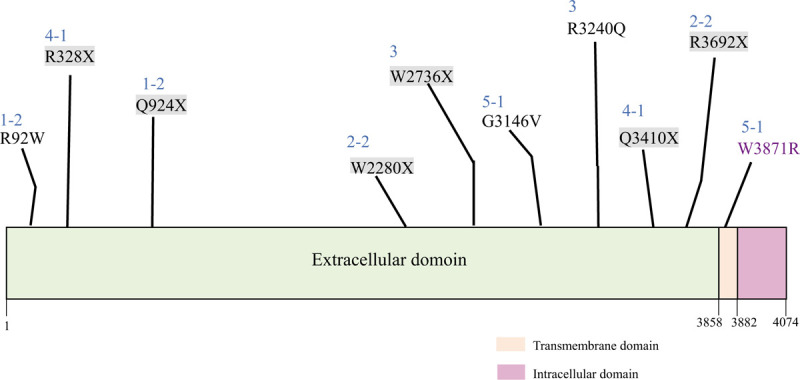
Schematic description of fibrocystin with indication of mutations found from our study. Domains of fibrocystin: green—extracellular domain, pink—transmembrane domain, violet—intracellular domain. Patient number is presented as blue numbers above each mutations. Nonsense mutations are indicated with gray highlight. Mutation from patient 5-1 was indicated with purple letter, because of the location different from other mutations. The schematic presentation was created by adopting the idea from Ren et al.^[16]^.

Pt 5-1 and 5-2 were female twins from the first pregnancy of the family. Oligohydramnios was first detected in both fetuses by US at GA 27 weeks (both AFI, <10 mm), with enlarged hyperechoic kidneys, leading to amnioinfusions for both fetuses. At birth, Apgar scores were 1 at 1 minute and 5 at 5 minutes for Pt 5-1, and 1 at 1 minute and 5 at 5 minutes for Pt 5-2, too. Both patients required immediate intubation and ventilator support, and soon after required HFOV because of pneumothorax, pneumomediastinum, and progressive respiratory failure. Pt 5-1 showed bilateral kidney enlargement with loss of normal corticomedullary differentiation (right kidney: 5.8 cm; left kidney: 5.8 cm) on kidney US, as well as hepatosplenomegaly and dysmorphic appearance of the lower extremities (club foot, clinodactyly of the right fourth and fifth toes). Pt 5-1 became anuric and died 18 hours after birth because of respiratory failure. Pt 5-2 also exhibited bilateral kidney enlargement with loss of normal corticomedullary differentiation (right kidney: 6.4 cm; left kidney: 6.5 cm) and mild pelviectasia of the right kidney (0.32 mm) on kidney US. Pt 5-2 also became anuric and died 15 hours after birth because of respiratory failure with recurrent pneumothorax. Peripheral leukocytes from Pt 5-1 were used for genetic analysis of *PKHD1*, revealing a novel mutation, c.9437G>T (p.Gly3146Val), in exon 58, and a known mutation, c.11611T>C (p.Trp387Arg), in exon 65. In silico investigation predicted that both mutations have damaging effects on the protein function (Table 2). The mother of the patients was a heterozygous carrier of c.11611T>C (p.Trp387Arg), and the father was a heterozygous carrier of c.9437G>T (p.Gly3146Val). The mutations found from 5 families are described in Fig. 2 by their location in the fibrocystin structure.

**Table 2:**
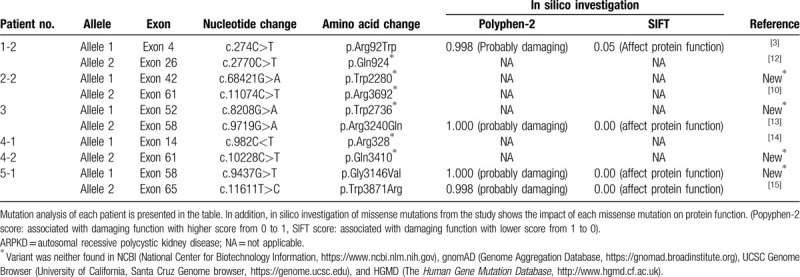
Mutation spectrum in patients with severe ARPKD.

## 4 Discussion

The current report describes the detailed clinical and genetic features of 9 fetuses or neonates severely affected by ARPKD, most of whom did not survive more than a few days after birth. The presentation of ARPKD was quite similar among our patients. The condition was suspected based on abnormal prenatal US findings, including enlarged kidneys with increased echogenicity (due to multiple microscopic cysts and loss of corticomedullary differentiation) between the second and third trimesters, which are the most typical findings in ARPKD.^[4,5]^ Oligohydramnios developed due to poor fetal urine output, requiring amnioinfusion for pulmonary maturation and survival, which was conducted in 7 of our patients.^[17]^ Renal anomalies with oligohydramnios were an important clinical sign for diagnosis.

The causative gene, *PKHD1*, is one of the largest genes associated with morbidity in the human genome.^[18]^ To date, >750 *PKHD1* mutations have been identified along the entire coding region (http://www.humgen.rwth-aachen.de). In our patients, a total of 6 truncating mutations (all nonsense) and 4 missense mutations were found in a compound heterozygous state. Three of the nonsense (p.Trp2280∗, p.Trp2736∗, and p.Gln3410∗), and one of the missense (p.Gly3146Val), mutations have not been reported previously, and were not detected in a control population screened from NCBI (National Center for Biotechnology Information, https://www.ncbi.nlm.nih.gov), gnomAD (Genome Aggregation Database, https://gnomad.broadinstitute.org), UCSC Genome Browser (University of California, Santa Cruz Genome browser, https://genome.ucsc.edu), and HGMD (The *Human Gene Mutation Database*, http://www.hgmd.cf.ac.uk). In silico analysis predicted that 4 missense mutations, including one novel mutation (p.Gly3146Val), would notably alter protein function (Table 2).

The protein encoded by *PKHD1*, fibrocystin, consists of a signal peptide (amino acids (aa) 1–23); a highly glycosylated, N-terminal extracellular region (aa 24–3858); a single transmembrane domain (aa 3859–3881); and a short cytoplasmic tail (aa 3882–4074).^[6,7]^ In the N-terminal extracellular region, there are several TIG/IPT domains (immunoglobulin-like folds shared by plexins and transcription factors), which regulate cell-to-cell adhesion and proliferation, as well as multiple PbH1 (parallel beta-helix 1) repeats, which may bind to glycoproteins on the cell membrane. The C-terminal cytoplasmic tail serves as a ciliary targeting signal.^[11,19]^

The full-length transcript is required for proper fibrocystin function, and a minimal critical amount of full-length functional protein may be required for normal tubular differentiation and maintenance of tubular architecture.^[11,20]^ Although it is difficult to characterize the functional effect of a particular genetic alteration, mutation type has been suggested to be associated with clinical severity in ARPKD; patients with 2 truncating mutations have a lethal phenotype, whereas the presence of at least 1 missense mutation may attenuate disease severity, acting as a hypomorphic allele that generates a partially functional protein.^[9,21]^ In our study, truncating mutations were associated with fetal or early neonatal death; however, the 4 patients from 3 families who carried missense mutations also exhibited severe phenotypes. Some missense mutations have previously been reported to result in severe phenotypes when accompanied by another allele encoding a truncating mutation, or when present in the homozygous state^[21]^; notably, the clinical course of the 2 patients in Family 5 with compound heterozygous missense mutations was also severe.

Differences in phenotypic severity among patients with missense mutations may be attributable to the location of the missense mutation, where this could influence glycosylation, structural stability, or interaction of fibrocystin with other molecules. Among the 4 missense mutations found in our patients, p.Arg92Trp is located in the first IPT domain; p.Gly3146Val and p.Arg3240Gln are both in the extracellular domain, which connects parallel β-helix repeats and the transmembrane domain; and p.Trp3871Arg is in the transmembrane domain. In addition, the conservation of each amino acid among different species can reflect the severity of missense mutations.^[13,22]^ Epigenetic changes, such as transcriptional modification, may also contribute to patient phenotypes.^[23,24]^

There are some limitations of this study. Our case series included only the most severely affected Korean neonates or fetuses, which do not represent the overall phenotypic and genetic features and their relationship of ARPKD. Due to the small number of cases, the pathogenic role of each domain of fibrocystin in the development of ARPDK was not able to be assessed.

In conclusion, our study presents 9 patients severely affected with ARPKD from the prenatal period, resulting in devastating outcomes, with discussion of their genetic alterations. Our observation of the same critical phenotypes in each family indicates strong genotype–phenotype relationships, with little intra-familial heterogeneity in ARPKD. The information generated in this study is important to inform reproductive planning in affected families.

## Acknowledgments

The authors are grateful to the patients and their families for participating in this study.

## Author contributions

**Data curation:** Jiwon Jung, Go Hun Seo, Yoo-Mi Kim, Young Mi Han, Ji Kwon Park, Jo Hoon Lee, Byong Sop Lee, Ellen Ai-Rhan Kim, Gu-Hwan Kim.

**Writing – original draft:** Jiwon Jung, Gu-Hwan Kim, Beom Hee Lee.

**Writing – review & editing:** Yeong Seo Park, Beom Hee Lee.
